# Updates on the Methodological Approaches for Carrying Out an In-Depth Study of the Cardiac Conduction System and the Autonomic Nervous System of Victims of Sudden Unexplained Fetal and Infant Death

**DOI:** 10.3389/fmed.2016.00054

**Published:** 2016-11-21

**Authors:** Graziella Alfonsi, Marina Crippa

**Affiliations:** ^1^Department of Biomedical, Surgical and Dental Sciences, “Lino Rossi” Research Center for the Study and Prevention of Unexpected Perinatal Death and SIDS, University of Milan, Milan, Italy; ^2^Service of Laboratory Medicine, Section of Pathological Anatomy, Department of Emergency, Clinica San Carlo, Paderno Dugnano, Italy

**Keywords:** SIDS, SIUDS, cardiac conduction system, brainstem, technical protocol

## Abstract

This article contains a set of protocols for histopathological techniques that can be used for carrying out in-depth studies of cases of sudden infant death syndrome and sudden intrauterine unexplained fetal death syndrome. In order to enable researchers to advance hypotheses regarding the causes of the unexpected death of infants and fetuses, the authors propose three innovative and accurate methodologies for studying the cardiac conduction system, the peripheral cardiac nervous system, and the central autonomic nervous system. Over the years, these protocols have been developed, modified, and improved on a vast number of cases which has enabled pathologists to carry out the microscopic analyses of the structures which regulate life, in order to highlight all the possible morphological substrates of pathophysiological mechanisms that may underlie these syndromes.

In memory of our research professor Lino Rossi (1923–2004).

## Introduction

In 1987, the first case of sudden infant death syndrome (SIDS) was delivered to the Institute of Pathology, University of Milan, Italy, as an object of study.

Since then, our researchers have written a large number of scientific articles and books, and held conferences and training courses on SIDS and sudden intrauterine unexpected fetal death syndrome (SIUDS) (1, 2).

Most of their research has been based on histological slides analysis, carried out with histopathological techniques that were intentionally and progressively designed to study the reasons behind the sudden deaths of the young victims.

The closure of the Institute of Pathology, the foundation of the “Lino Rossi” Research Center for the study and the prevention of unexpected perinatal death and SIDS, University of Milan, as well as the enactment of Italian law No. 31 of February 2, 2006 have led us to examine a considerable number of cases, including control cases, in order to monitor the protocols continually.

## Materials and Methods

The diagnostic protocol for carrying out anatomic–pathological and forensic–medical investigations on SIDS and SIUDS victims during the last 3 months of pregnancy includes three investigation procedures:

1. The anatomic–pathological investigation.
2. The molecular genetics investigation.
3. The toxicological investigation.

All the technical protocols used for this study strictly concern anatomic–pathological investigation (3, 4).

For each case, it is essential to have family medical records, documents stating the place of death (in the event of SIDS), the informed consent of the victim’s parents, as well as the anatomical samples required for carrying out the investigations.

Downloadable forms are available online: http://users.unimi.it/centrolinorossi/en/protocollo_diagnostico.html.

The anatomical samples required specifically for the study are:

- the thoracic block, composed of both lungs, larynx, trachea, esophagus, thymus, the whole heart, within the pericardial sac, and part of the diaphragm;
- encephalon;
- thoracic spinal medulla.

According to Title X° of Italian D. Lgs 81/08, “Behavioural Rules in Biological Laboratories,” the anatomical samples must be immersed in 10% neutral-buffered formalin solution, placed in a hermetic plastic container, in order to avoid operator risks, and marked with patient identifiable information and the medical record number of the hospital in which the case originated.

When a case is accepted, the doctor checks both the data and anatomical samples provided and dictates a description of the organs to the technician.

After full fixation, the doctor dissects the organs: he cuts standard samples, that will be processed and treated according to routine histological techniques (5–8), as well as samples for carrying out specialized diagnostic analyses and research procedures (9–12).

### Peculiar Specimens to Be Taken

The specimens (13–15) excised specifically for studying the cardiac conduction system (CCS) are:

- sinatrial (SA) node;
- atrioventricular (AV) system.

The specimens (16) required for studying the autonomic cardiorespiratory nervous system are:

- mediastinal plexuses (coronary, intertruncal);
- intercarotid receptors;
- cervical sympathetic ganglia (stellate ganglion);
- thoracic spinal medulla (T1–T5);
- samples of brainstem.

In the event of SIUDS, fetal adnexa may be observed, both in anatomical samples and on histological slides.

The specimens excised for fetal adnexa are:

- one roll of free amniochorionic membrane (two rolls, if infection is suspected);
- three segments of umbilical cord;
- the area below the umbilical cord;
- the main ramifications of amniochorionic vessels;
- a macroscopically undamaged cotyledon from the third central placental disk;
- all areas that the anatomic-pathologist considers to be of interest;
- all changes in caliber and shape of the amniochorionic and funicle vessels (17).

As for routine samples, these samples are processed with an automatic processor.

## Stepwise Procedures

### The Basic Histological Techniques

There are fundamental steps that a technician should take in order to obtain optimal outcomes (18).

The basic steps are as follows.

1. *Fixation*: this step is required to prevent postmortem changes such as autolysis and putrefaction. For organs obtained from an anatomy theater, the most used fixative is 10% neutral-buffered formalin solution (in phosphate buffer), commercially produced, due to the risks involved at maximum concentration (H311, H331, H351, H371, H314, and H317).
2. *The pathologist’s sampling methods*: according to an established protocol, it is essential to cut the organs into thin (4 mm thick) slices, so that the fixative will penetrate the tissue within a reasonably short time.
3. *Post-fixation*: after sampling, it is essential to change the old formalin with new formalin.
4. *Processing*: i.e., the anatomic sample undergoes a sequence of graduated transformations, to replace the water contained in the tissue with hardener medium, in order to give support and solidity to the sample itself. Various histopathological methods can be applied for this purpose. In a routine histological technique, this step is carried out by an automated routine tissue processor, which is able to process samples automatically in time periods that range from 2 to 17 h, in relation to the size of the samples (the bigger the sample, the longer the processing time). At the same time, it is possible to process biopsies (minimum size 1 mm) and operating samples (3 mm maximum thickness), individually contained in small closed biocassettes.
5. *Embedding*: i.e., to make the sample into a geometrical shape, with a possible support (ring or cassette), which can be firmly fixed to the specimen holder of the microtome. Embedding is always carried out with the aid of a device, the complete embedding center, which dispenses hot paraffin (56–58°C) and subsequently cools the block, placed into standard base mold.
6. *Cutting with the microtome*: the final sample is cut into 3 µm thick sections (1 µm = 1/1000 mm). The microtomes most widely used in diagnostic anatomic pathology laboratories are rotary, manual, or automatic microtomes that are very precise as they can cut extremely thin sections, while the most suitable microtome for the protocols described in this article is a steady and heavy sledge microtome, with spacers on the knife block, for cutting wider than standard blocks, as some of the blocks are over 4 cm wide (standard thickness is 0.3–0.5 cm).
7. *Extension of the sections*: the thin sections cut with the microtome are immersed in a flotation bath, containing water heated to 37°C, to aid extension, and they are then placed on port-object slides, countermarked with the case number and the number of the specimen. The slides are then placed into a 37°C oven, overnight, to improve the adherence of the section to port-object slides [it is also advisable to add chemical adhesive [FIX-ON, Kaltek (PD), code 0872] to the water in the flotation bath]. The drying time of a histological slide can be shortened by placing the slide in oven at 60°C, for 30–60 min.
8. *Staining*: the sections obtained must undergo “staining” procedures for viewing them under a microscope. The various staining procedures are histological staining (to see the morphology of the sample), histochemical staining (to see particular components of various tissues), and immuno-histochemical staining (when a specific cytoplasmic and/or nuclear component of the tissue or the cell must be branded). In routine procedures, technicians generally use histostainers and immune-histostainers which have numerous colorations and reactions, but not all. There are also automatic devices for mounting slides, instead of technicians.

As a matter of routine, it is essential to standardize the samples as much as possible and use several types of scientific equipment in order to speed up the setup time of the histological slides. A histological/histopathological slide can be ready in about 2 days.

### Technical Protocol

The peculiarity of this technical research, for studying the CCS and the autonomic central and peripheral nervous system, is that the tissue components are not macroscopically visible, and they have a tridimensional development, therefore, in order to pinpoint and study them, it is essential to cut the blocks with the microtome for most or all of the block of the research.

This is known as the “seriation” technique, which is carried out using a microtome.

**Figure 1:**
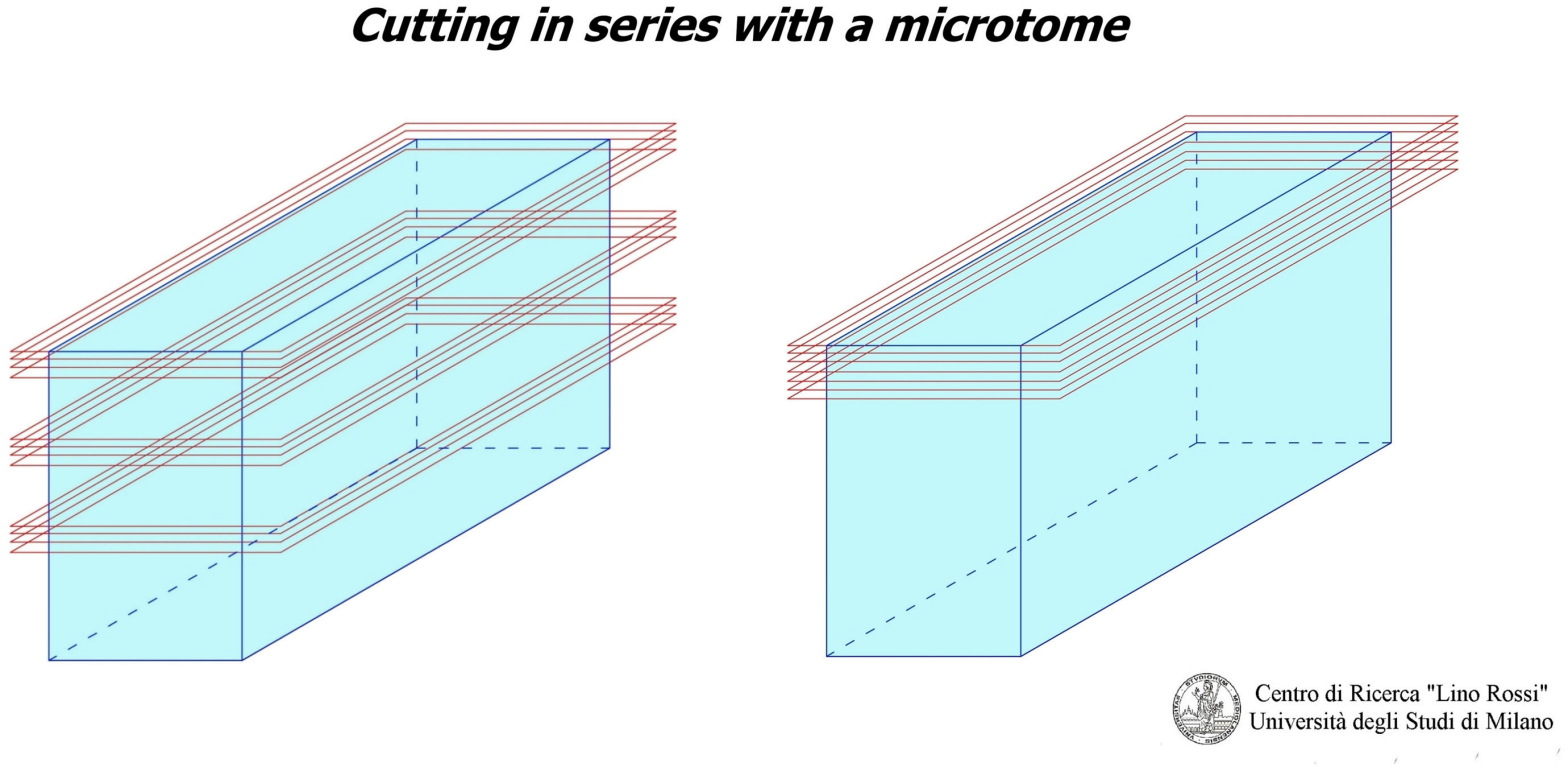
**There are two seriation techniques**.

As you can see in Figure 1, there are two “seriation” techniques:

1. Seriation at prearranged intervals: i.e., when the technician discards anatomic matter between one level and next, which is the method used for the CCS.
2. Continual seriation: i.e., the technician slices extremely thin sections (only a few microns thick) one after the other, without prearranged intervals, which is the method used for the central and peripheral nervous system.

At the frosted-end of the port-object slide, the technician writes the case number, the sample number, the level number, and the sequence of the section in the level.

### Technical Protocol for Studying the Cardiac Conduction System

The tissue block sampling method for studying the CCS was developed by Professor Lino Rossi in 1955 (AV system) and in 1958 (SA). The technical procedure is described in the appendix of his book “Arrhytmologic Pathology of Sudden Cardiac Death” (19).

Professor Lino Rossi left his technical methodology to the authors who “have correctly interpreted his expectations” in over 20 years of work experience and are capable of speaking and writing on these topics (20).

The first block, SA, “includes the sinoatrial node, its atrial approaches,” the terminal crest, and the sinoatrial node ganglial plexus (21).

**Figure 2:**
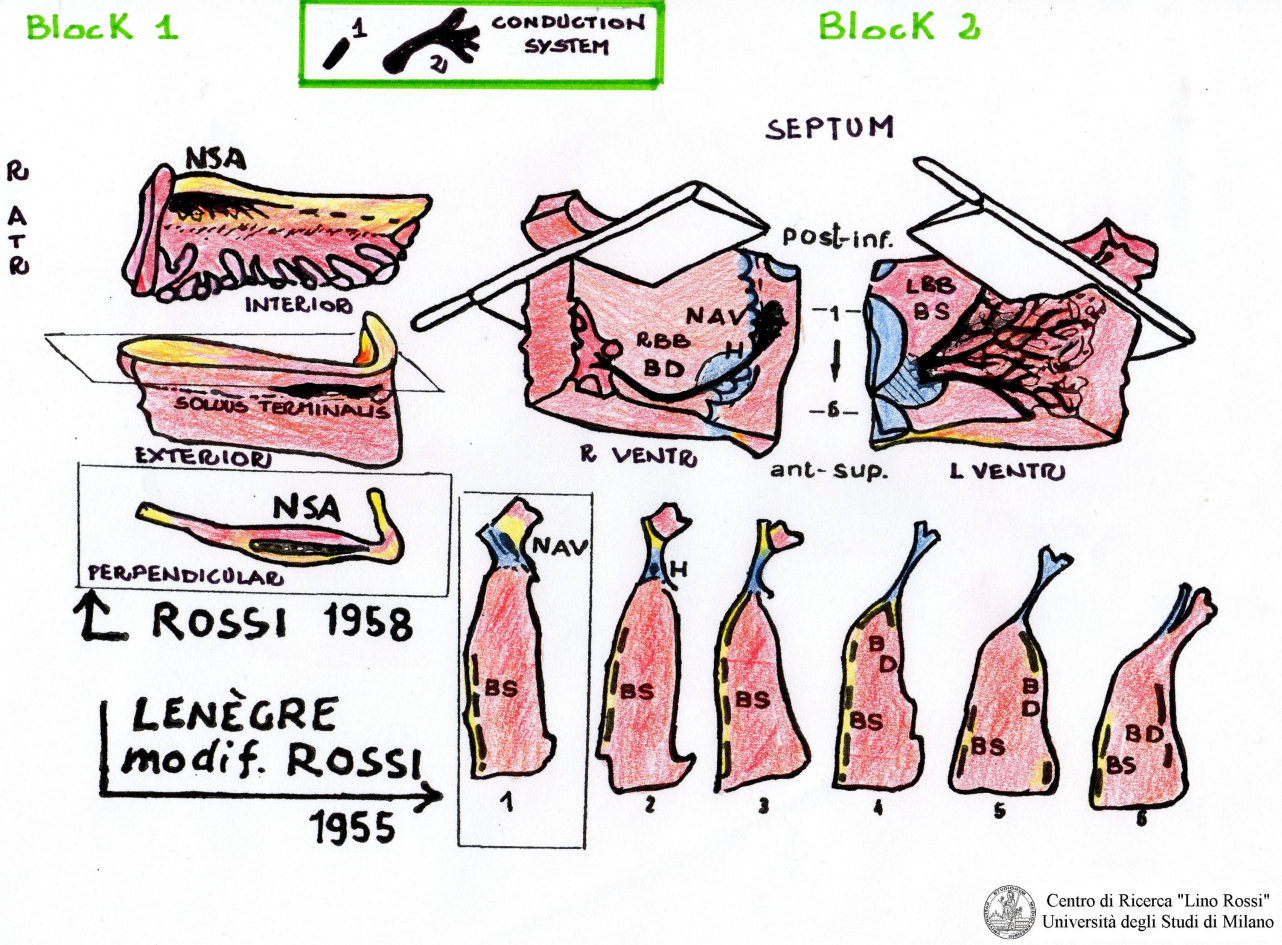
**Sampling and series of the cardiac conduction system by Lino Rossi**.

The second block, AV, includes the AV system and its atrial approaches, as shown in Figure 2.

The two blocks, such as SA and AV, will be partially reduced by excising redundant portions (papillary muscles, chordae tendinae, etc.), which will be post-fixed in 10% phosphate-buffered formalin.

With the aim of reducing the number of histological sections, some researchers cut these two blocks into five to six large, parallel slices (22).

The large slices are then processed and embedded by hand, or by means of automatic processors.

The slices of interest can be cut in series with the microtome.

This method is definitely faster even if less accurate than the method developed by Professor Rossi as anatomical material can be lost and the spatial orientation of the structures of interest can be hard to define.

Once fixation is complete (3–14 days according to specimen volume): in order to simplify this process, it has been summarized as follows.

All of the following steps are performed manually, because these samples are very long and wider than routine samples.

The samples must be rinsed under running water for approximately 20 min in order to remove the formalin. The tissue is transferred to 95% ethanol for 24 h and immersed in pure 1,4-dioxane (diethylene dioxide) four times for 12 h each treatment, for dehydration and clarification.

**Figure 3:**
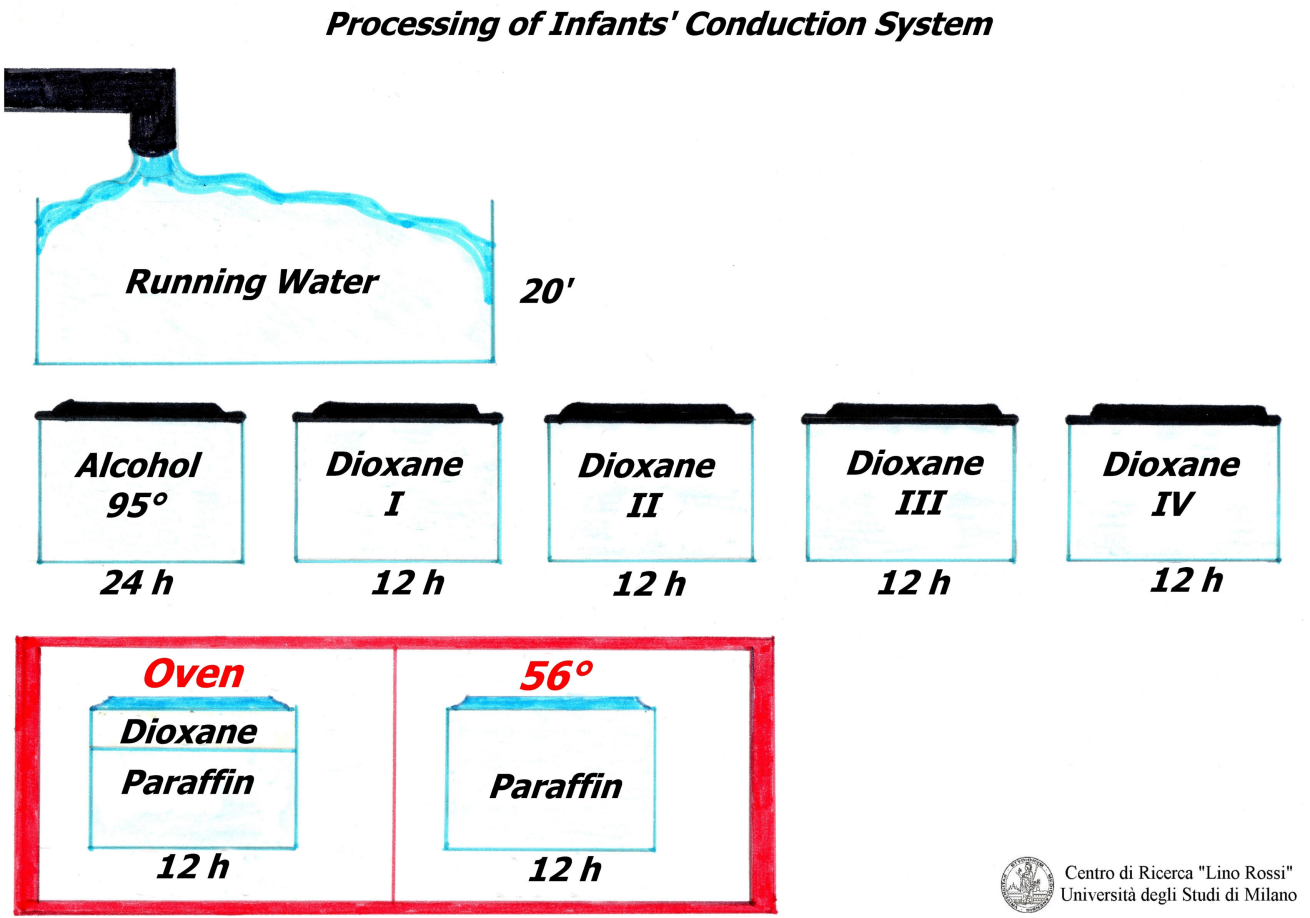
**Processing of the cardiac conduction system**.

The samples are then partially impregnated in 1/3 dioxane-2/3 paraffin (fusion point 56–58°C) and then totally impregnated in pure paraffin (fusion point 56–58°C), in oven (Figure 3).

Professor Lino Rossi made a wise decision by using dioxane as it is both a dehydrator and a paraffin solvent and is therefore essential for this conduction system technique: on the other hand, if the routine ethylic dehydration method is used, the material tends to harden, resulting in breakages during microtome sectioning.

In 1931, Graupner and Weissberger introduced successfully dioxane; the only drawback being that it costs approximately three times more than ethanol.

Following the dehydration process, tissue embedding in paraffin is carried out with metal molds (Leuckhart’s “L” pieces), if the specimens are oversized.

The same technique is used for new-born babies and fetuses, although at times it is difficult to excise the SA and AV blocks in fetuses of low gestational age.

A whole fetal heart can be embedded with this protocol, taking care to extend the process phases.

The inclusion must be carefully oriented.

**Figure 4:**
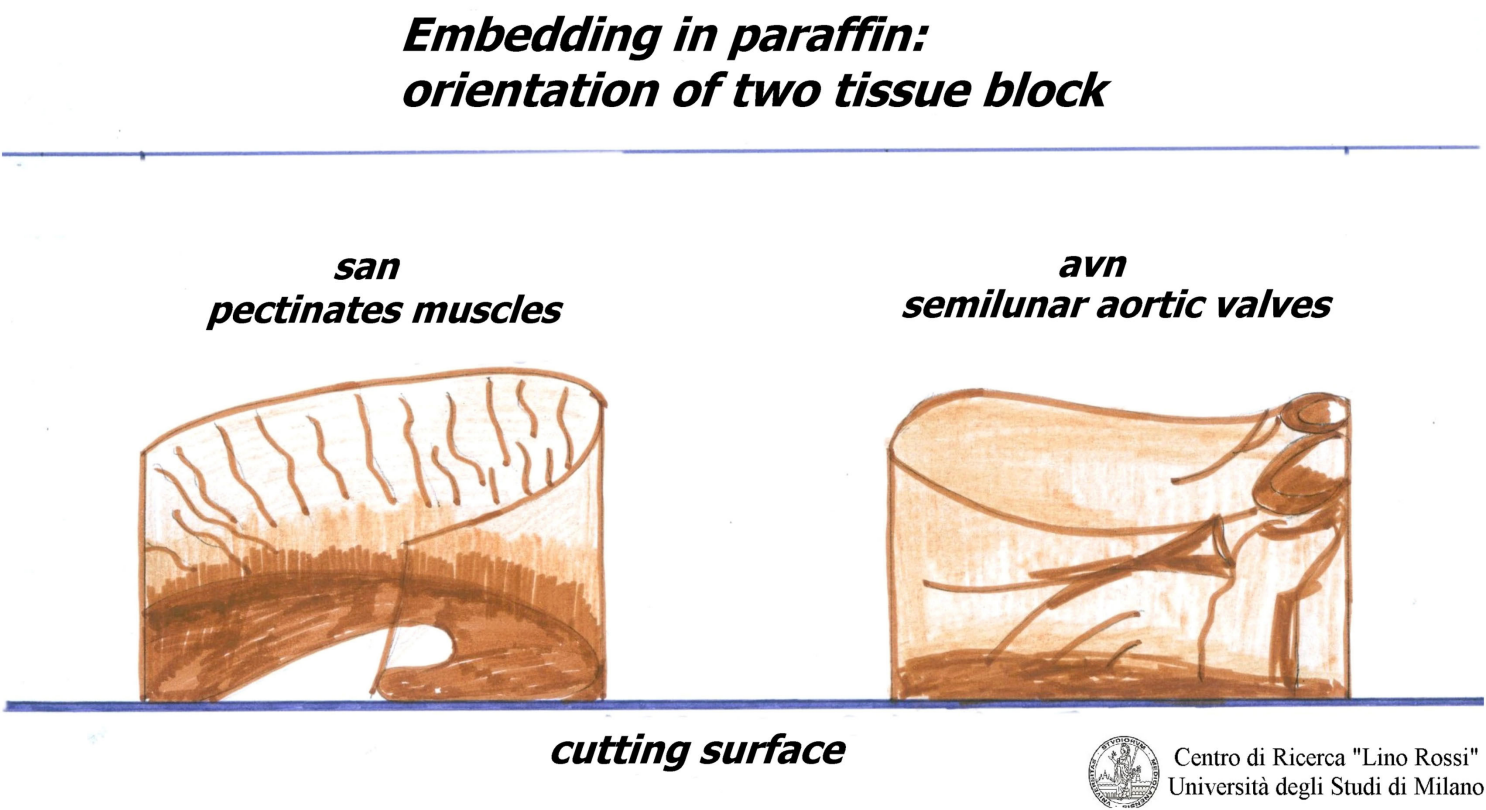
**Embedding of SA and AV**.

For the SA node, it is essential that the pectinates muscles point upwards, as shown in Figure 4.

For the AVS, the aortic semilunar valves should be placed opposite the point of incidence of the cut.

The blocks must be stored in a refrigerator.

**Figure 5:**
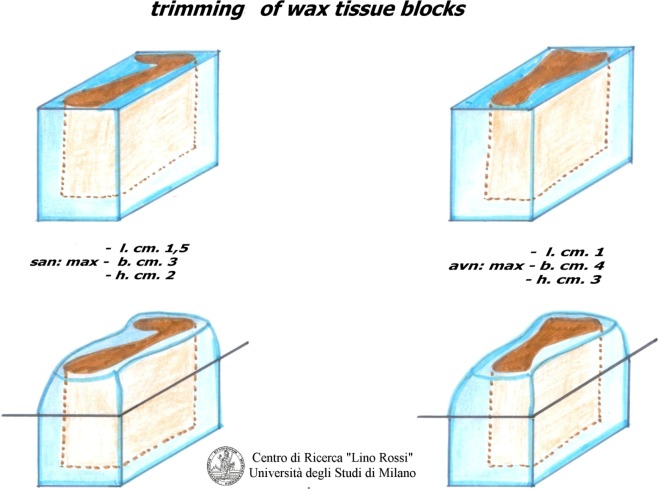
**Trimming of the blocks**.

As shown in Figure 5, the surface paraffin is previously removed from each inclusion, and molded, around the specimen with a scalpel in order to facilitate cutting with a microtome and to improve its distension in warm water.

It is advisable to make the inclusion fairly high up, so that it can be firmly fastened into the holder of the microtome; wood or plastic supports should be avoided as they tend to come off.

As already mentioned, the serial section is essential for investigating conduction system as it allows for a three-dimensional reconstruction of the AV tissue formation under study.

**Figure 6:**
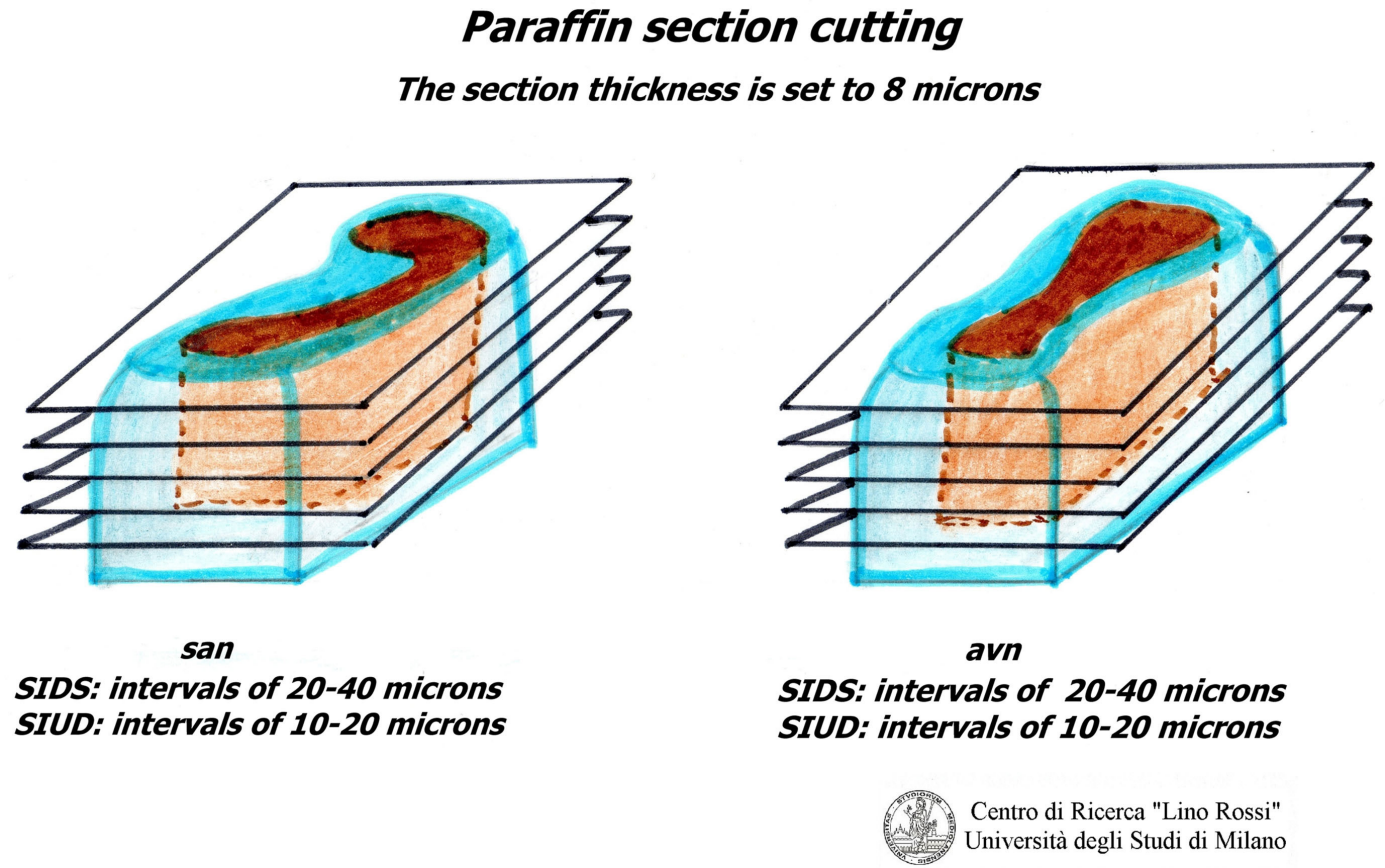
**Cutting of the blocks**.

Due to the dimension of the specimen, it is advisable to carry out intercalated section every 20–40 μm interval, thus collecting three 8 μm sections at individual level; it would be rather time-consuming to carry out a global serial section of the entire specimen on a routine basis in addition to the work and material (Figure 6).

Following the microscopic control of the slides, one can decide whether to terminate the analysis. In this case, the residual inclusion could be stored for further control.

The serial sections can only be carried out with normal manual sledge microtomes, adapted for tall specimens.

The sections obtained are then distended in 37–40°C warm water, in a flotation bath, containing a chemical adhesive.

The sections for histochemical analysis are collected on clean frosted-end slides, while the sections for immunohistochemistry are placed on slides pretreated with 3-aminopropyltriethoxysilano (silanized) (Sigma-Aldrich, code A 3648).

Both types of slides must be marked with the case number, the inclusion number and the level sequence number, with “silanized” or “unsilanized” indicated on the slide which is, then placed in an oven heated to 37°C and left overnight.

The slides, to be stained for apoptosis (Apoptosis detection kit, Chemicon International, Temecula, CA, USA, code S 7101), must be put back into the oven heated to 60°C for 4 h in order to improve the adherence of the finely cut tissue into the glass slide, which is of great importance.

**Figure 7:**
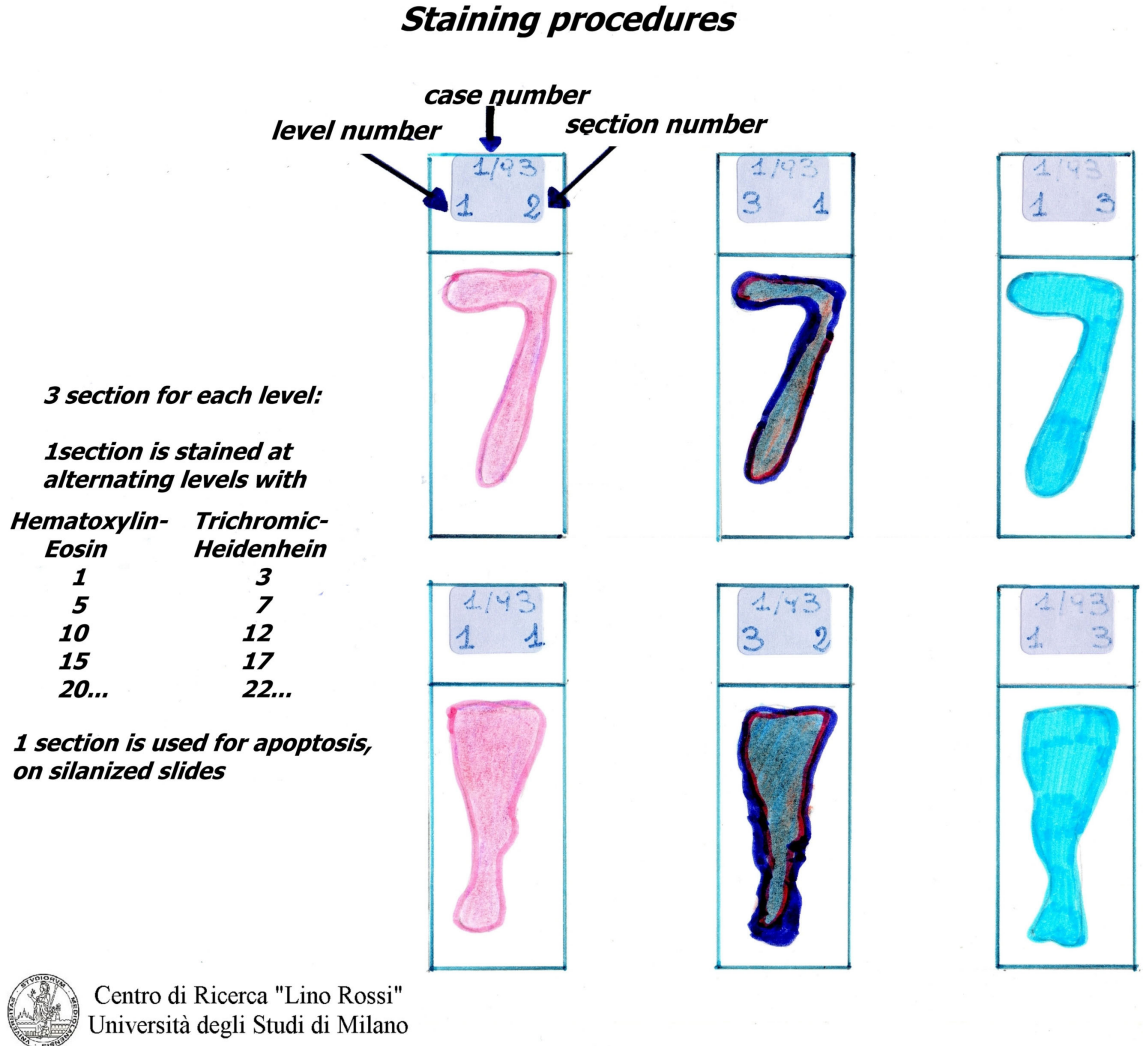
**Routine staining of the CCS**.

It is not necessary to stain all of the level slides (for SA 70-100, for AV 100-130) in order to prescreen. It is sufficient to select specimens every five levels and stain them using the hematoxylin–eosin (H&E) and Heidenhain staining method for connective tissue (AZAN) alternately (Figure 7).

Hematoxylin–eosin is probably the most widely used histomorphological staining procedure. This staining method is able to demonstrate an enormous number of different tissue structures, including conduction system structures: the hematoxylin stains the cell nuclei blue, and outlines the nuclear details, while eosin stains cell cytoplasms and connective tissue fibers various shades of pink and orange.

The stains are made in our laboratory: Mayer’s hematoxylin, an alum hematoxylin, chemically ripened with sodium iodate, and eosin 0.5%, both in aqueous solutions.

The second, Heidenhain’s AZAN (1916), is a variant of Mallory’s method. It is a trichromic staining procedure for connective and muscular tissues. This technique stains nuclei and erythrocytes red, muscles orange–red, and collagen various shades of blue.

The term AZAN is derived from the first syllables of AZocarmin G (Color Index 50085, Sigma-Aldrich, code A-1091) and ANilinblue water soluble (Color Index 42755, VWR International, code ACRO 229661000)-Orange G (Color Index 16230, Carlo Erba Reagents, code no. 13), which are the names of the principal dyes used.

In order to obtain good results, one should overstain the myofibril conducting muscle with azocarmin, preheated in oven at 55–56°C, and differentiate quickly with anilin oil, thus blocking it with diluted acetic acid and then mordanting with phosphotungstic acid before staining in anilinblue-orange G (for a combination) (23).

**Figure 8:**
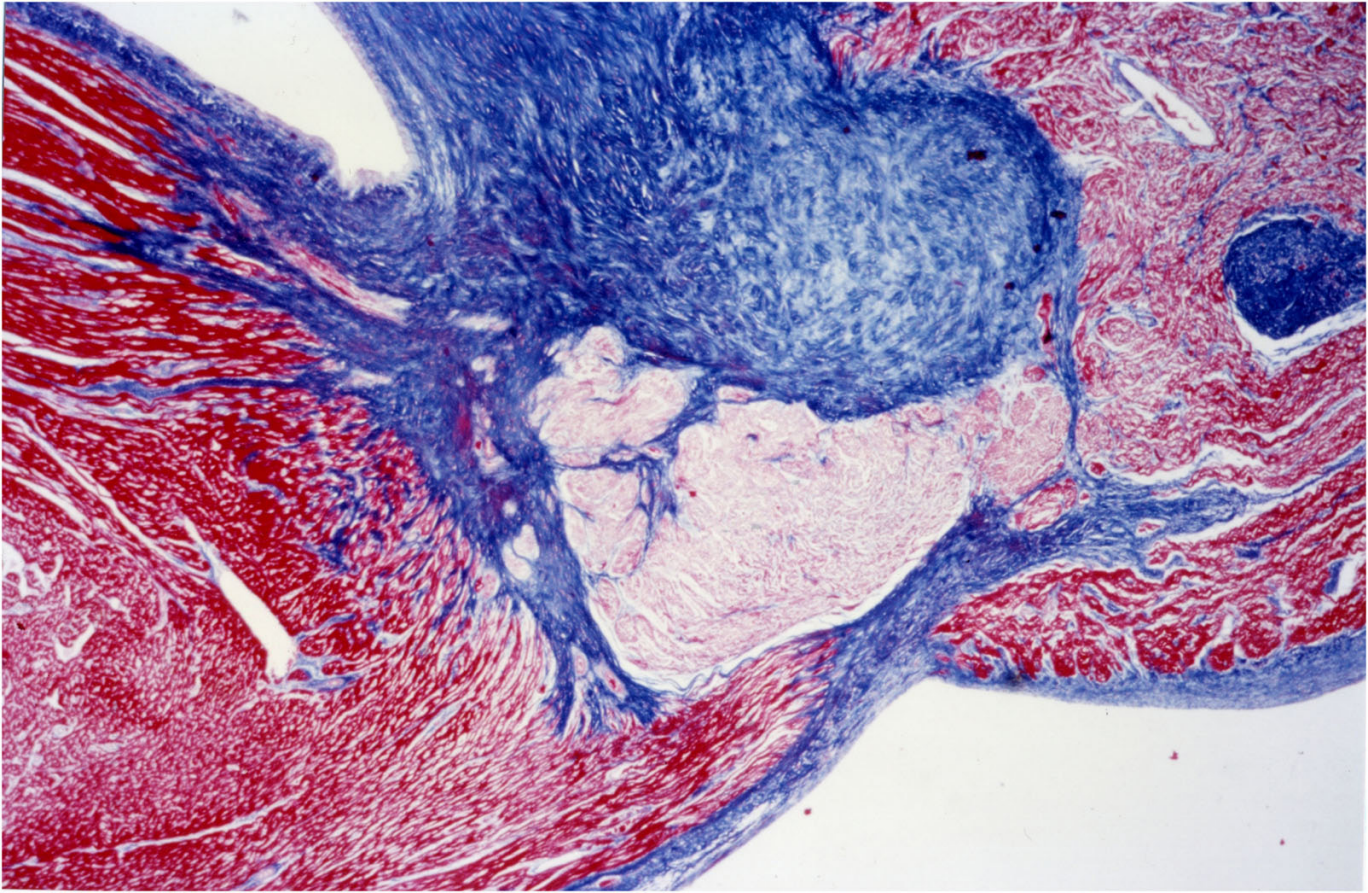
**AV system, His bundle: staining difference between common myocardium and specific myocardium, AZAN, 4×**.

Heidenhain’s trichrome clearly differentiates the common myocardium, which is colored with orange G in bright red, from the specific myocardial which appear pale, with the same dye, due to the presence of fewer contractile fibers, while the connective tissue assumes an anilinblue staining that is a more or less intense bright blue (Figure 8).

As for H&E, all the staining solutions of AZAN are made in the laboratory.

The blank slides are archived for carrying out follow ups of the investigations.

Depending on the abnormalities observed, it is possible to use blank slides for additional stainings.

The histochemical stainings, Periodic Acid Schiff (PAS) and Alcian Blue 8GX (Color Index 74240, Sigma-Aldrich, code 05500) at pH 2.5, are used to point out the cartilaginoid hypermetaplasia of the central fibrous body of the heart of infants who died of SIDS.

Alcian blue is also useful to highlight mast cells in tissue: cytoplasm deep azure with red nucleus in a light blue background.

According to Frank B. Mallory, following the modified method of Pathology Institute of American Armed Forces (A.F.I.P.), satisfactory results are obtained with Phosphotungstic Acid Hematoxylin (P.T.A.H.) instead of, or in association with AZAN.

**Figure 9:**
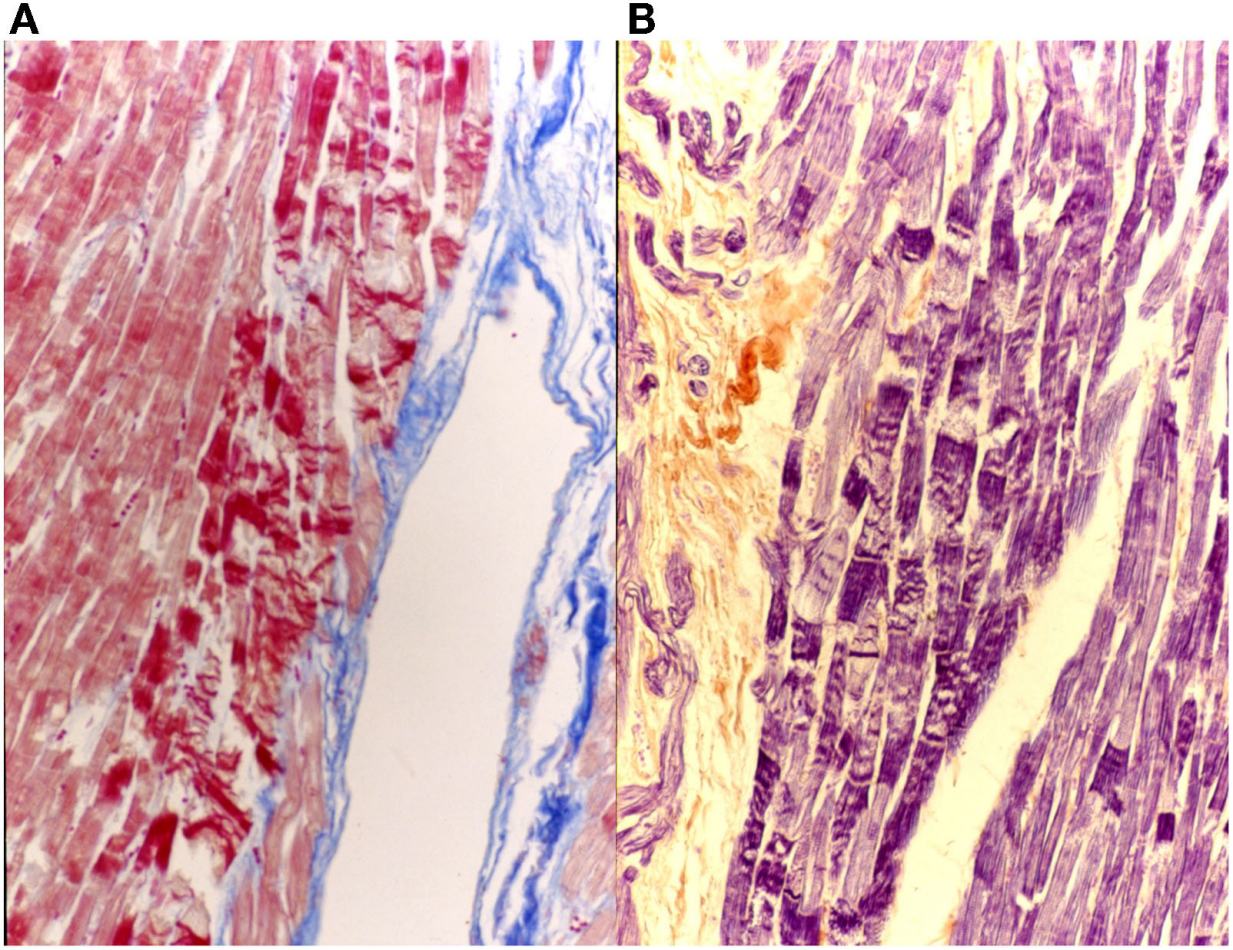
**Myocardial tissue suffering hyperacute ischemia (initial contraction bands)**. On the left **(A)**, AZAN 40×; on the right **(B)**, P.T.A.H 40×.

This staining is suitable for the central nervous system and the heart. The results of the staining are nuclei, fibrin, fibrils of neuroglia and the myoglia, the fibroglia and the contractile elements of striated myocardial and skeletal muscle blue; collagen, reticulum, elastin, cartilage and bone matrix are yellowish to brownish red (Figure 9).

Weigert’s method is suitable for staining elastic fibers black or deep brown, on a yellow-red background according to Van Gieson staining for gross connective and muscle tissue, yet it is seldom used as it is a contrast-staining and generally fades away after two or three years.

The Resorcin–Fuchsin method provides satisfactory results for elastic fibers (1898).

Good results were obtained with the “slow Eosin” (very diluted, a couple of drops in a glass of water, approximately 0.1%) for the differential staining of leukocytes in eosinophilic myocarditis.

The silanized blank sections may be suitable for immunohistochemistry such as proliferating cell nuclear antigen (PCNA) (Novocastra Reagents – Leica, code NCL-L.PCNA), Apoptosis (TUNEL assay), S 100A1 (Dako, code Z0311), c-Fos (Santa Cruz Biotechnology, code S52), Sarcomeric Actin, clone Alpha-Sr-1 (Dako, code no. M 0874) (24, 25).

### Technical Protocol for Studying the Peripheral Cardiac Nervous System

As far as the peripheral cardiopulmonary nervous system is concerned, adequately countersigned, mediastinic plexuses, intercarotid receptors, and stellate ganglia are fixed in 10% neutral-buffered formalin.

For infants and fetuses, it is advisable to carry out sampling on previously fixed material.

The sampling of medistinic plexuses consists in removing the fibroadipose tissue between the pulmonary artery and the aorta and between the aortic arch and the hilum of the lungs (intertruncal plexuses). The coronary plexus is in the roots of the coronary artery. In infant and in fetus sampling is limited.

The carotid body is located near the carotid bifurcation, beside the internal carotid, in the vicinity of the carotid sinus. The carotid body is a differentiated paragangliar organelle, while the carotid sinus is an anatomic area of the middle tunica of the artery, in which many nerve endings are present.

Sympathetic cervical superior ganglion is located near the intercarotid block. The middle sympathetic cervical ganglion is difficult to find, while the inferior sympathetic cervical ganglion is joined to the first thoracic ganglia to form the stellate ganglia, near the branch of the vertebral subclavian artery.

Samples should be thoroughly rinsed with running water immediately after fixation with 10% neutral-buffered formalin.

Regarding the peripheral cardiac nervous system, the brainstem and thoracic spinal cord, the dehydration of these tissues differs to that observed for the conduction system study. The ablated fragments have a soft consistency (fibro adipose tissue) therefore it is advisable to use a suitable dehydration to harden up the specimens. For this purpose, ethylic alcohol was used at increasing concentrations (70%, 95% in two changes, 100% in two changes), followed by xylene, in two changes, and embedding into two changes of pure paraffin at fusion point 56–58°C, in the oven.

The procedure used for the brainstem can also be used for these samples (as shown in Figure 12, below), but the samples are kept in the solvents for less time (1 h and half instead of 3 h). The second change in alcohol 95% is overnight, also for the second change of xylene.

It is advisable to make these changes manually since the dimension of the specimens may vary.

Embedding is then carried out following a precise protocol.

**Figure 10:**
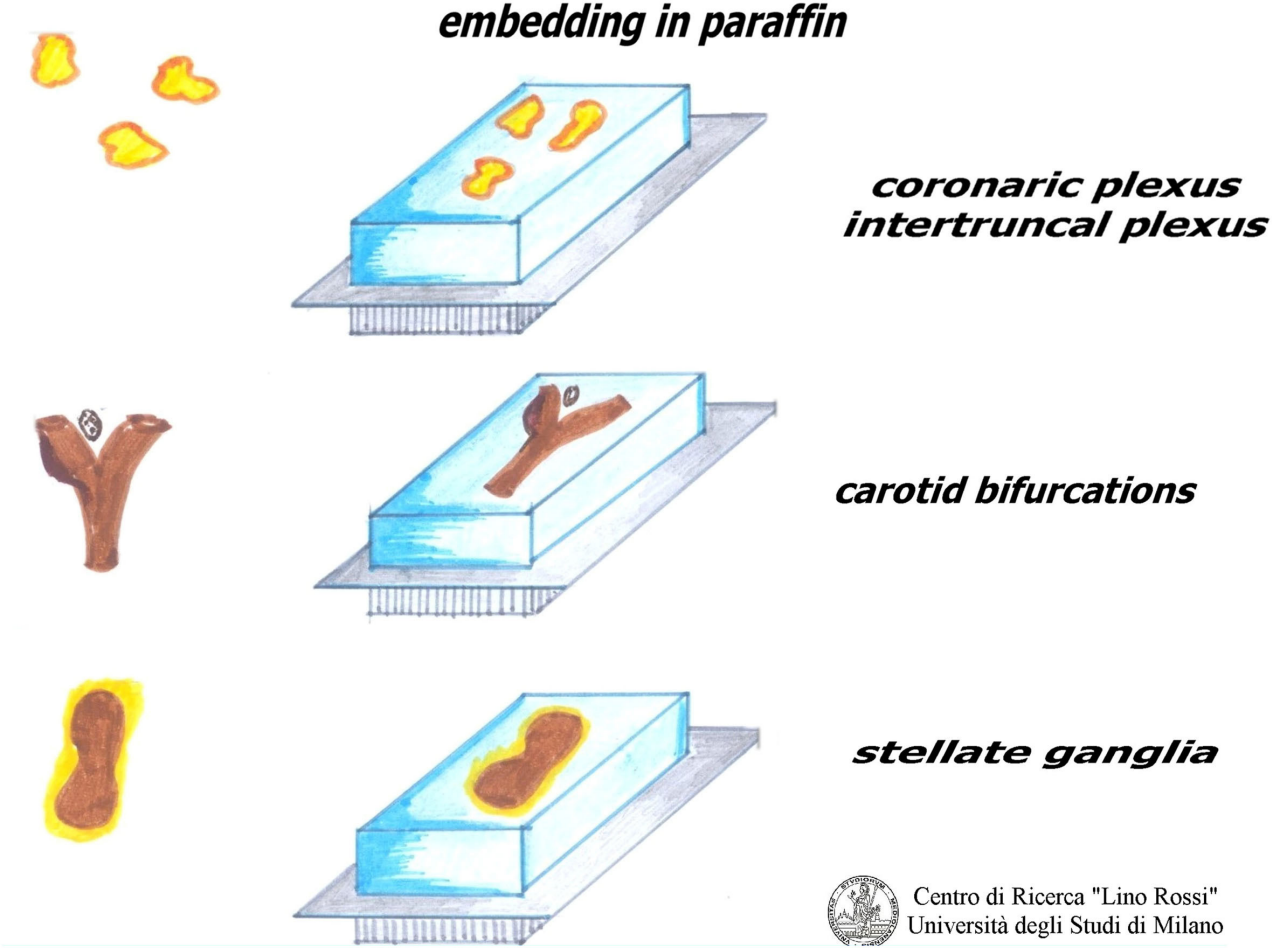
**Embedding peripheric cardiac nervous system**.

As shown in Figure 10, the plexuses must be embedded by pressing the fibroadipose tissue onto the cutting surface in order to visualize the maximal surface of each section; in this way, we are more likely to be able to see the microscopic nervous receptors.

The ganglia can be embedded whole, or can be subdivided according to length and analyzed together.

Only a flat embedding of the carotid bifurcation, right and left separately, provides a good view of specimens, since the carotid glomus is located between the two carotid branches, a little above the bifurcation, as the sinus is part of the internal carotid root.

The embedding of plexuses, ganglia, and bifurcations must be carried out with embedding rings, which are more stable than disposable cassettes, because these specimens are small in the fetuses and infants.

After cooling in a refrigerator, the upper paraffin layer should be taken off the blocks obtained and scalpel-trimmed in order to reduce the peripheral paraffin surplus surrounding them, thus facilitating section distension during microtome cutting.

The blocks must be cut into series, to allow for three-dimensional reconstruction and/or to increase the likelihood of finding the neuro-receptor, since these structures have numerous individual variants.

Conventionally, the serial sections at plexus, ganglia, and carotid bifurcation are six sections for each level, the individual sections are 5 μm thick (two for H.E. staining and four blanks), without intervals. The specimens should be cut whole.

Which is the best histologic staining technique?

The H&E as a screening, for everything.

An argyrophil silver technique, such as the Grimelius silver method, can be useful for demonstrating the granulations in the receptorial cells of the small ganglions present in the plexuses, the glomera and the ganglia.

These granulations are able to link with silver alogenures, which must be reduced with a reducing reagent, by transforming silver salt into metallic silver with a solution containing hydroquinone. The results show dark black granulations on a yellow gold background.

The electivity of Grimelius method is obtained by pre-treating silver salts in a 60°C solution, followed by a short 45°C re-impregnation which provides a further metal deposition, thus making the final slide more visible.

**Figure 11:**
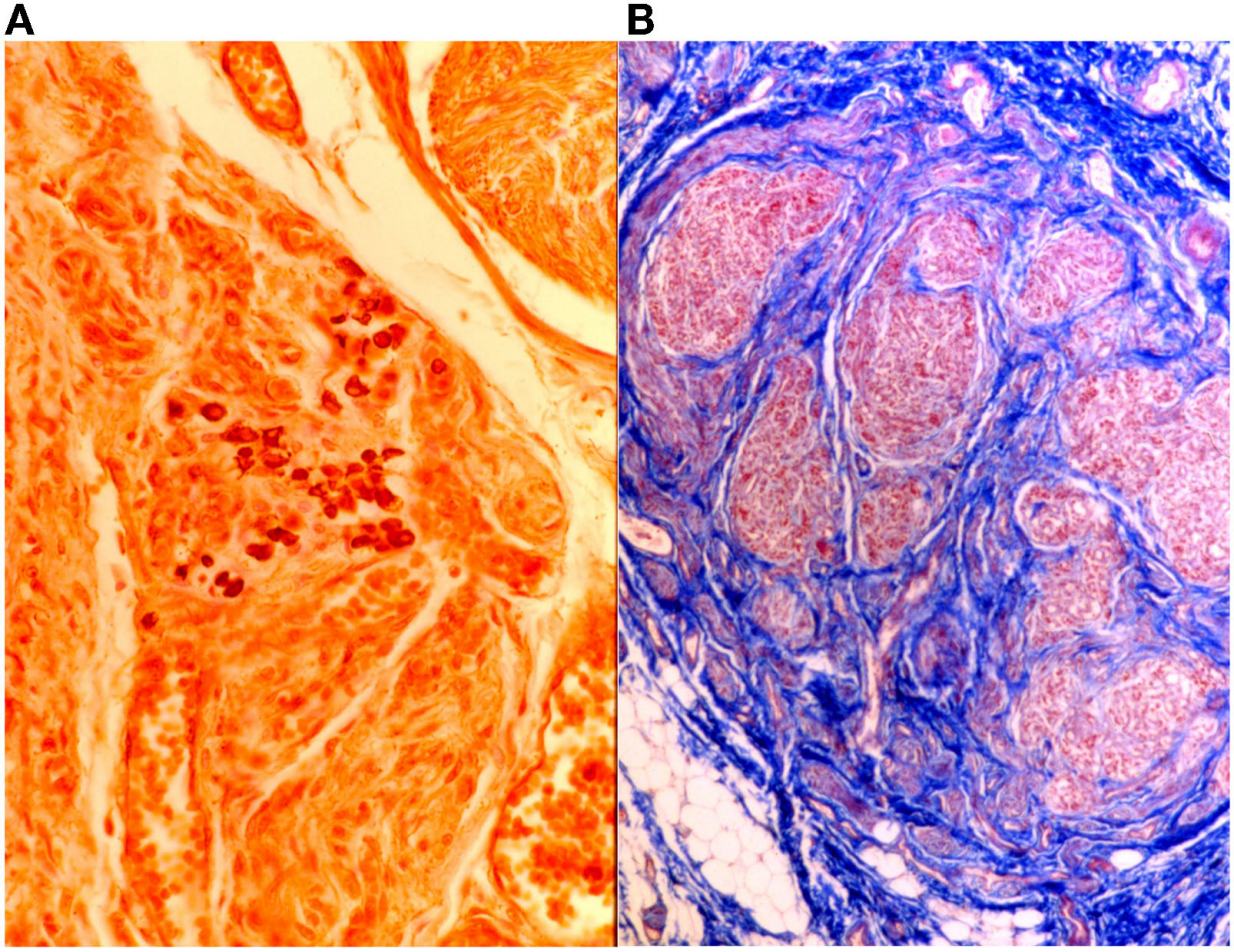
**On the left (A), intertruncal plexus with chief cells rich in cytoplasmic argyrophilic neurotransmitter granules, Grimelius, 40×; on the right (B), carotid body in AZAN, 25×**.

Heidenhain’s AZAN can also be used to highlight glomic neuroreceptors (Figure 11).

Over the last few years, few studies have been carried out on peripheral cardiac receptors due to the continual in-depth studies of the neuronal centers “of the human brainstem, involved in breathing control in perinatal life” (26).

### Technical Protocol for Studying the Brainstem and the Thoracic Spinal Cord

The sampling of the brainstem foresees the following four sections:

1. Third lower midbrain – third upper of pons (embedded from pons).
2. Intermediate pons (not to be seriated) (embedded from caudal surface).
3. Third lower pons – third upper brainstem (embedded from pons).
4. Obex (embedded cranial surface).

**Figure 12:**
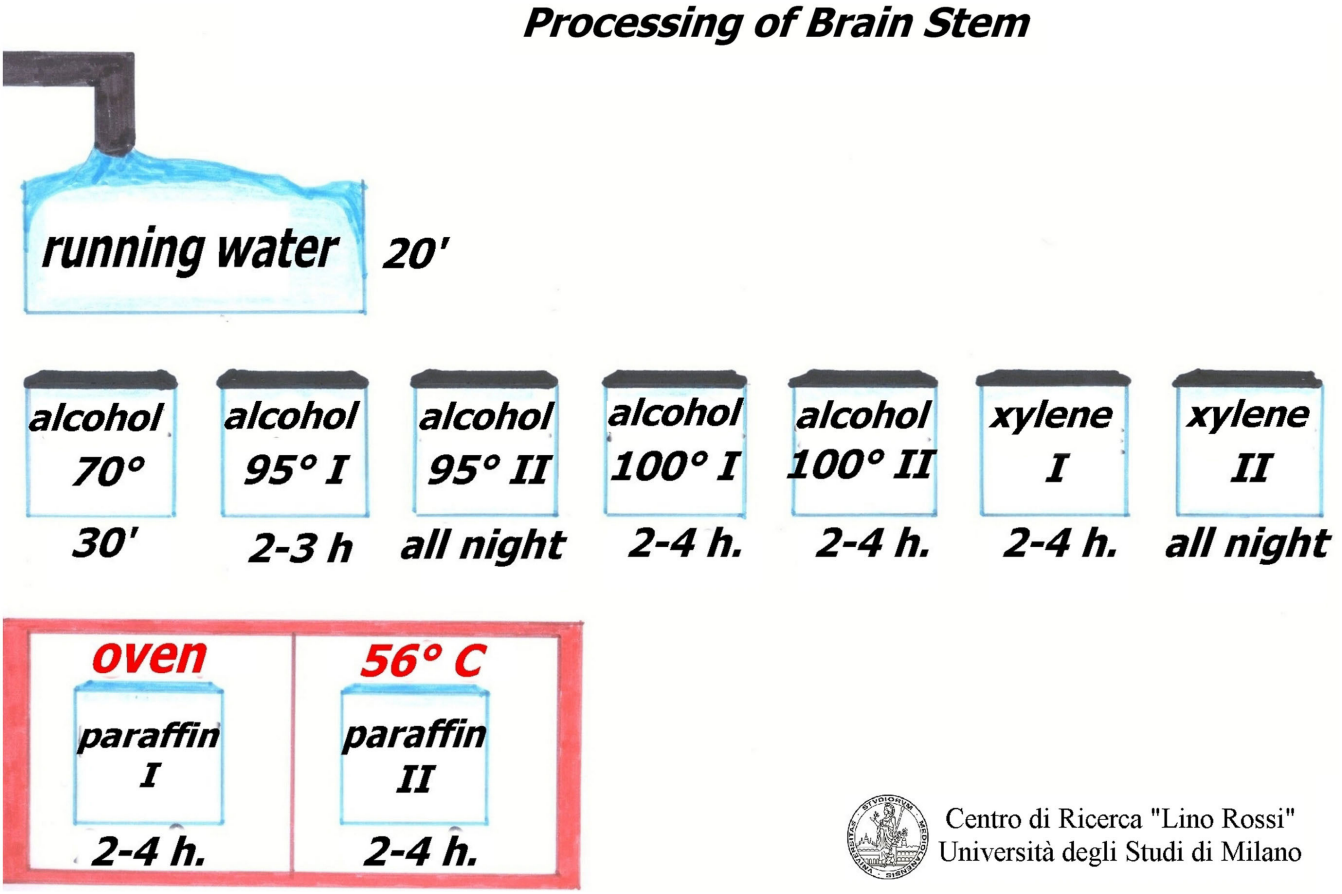
**Processing brainstem and thoracic spinal cord**.

Figure 12 shows the manual processing of these samples.

After fixation in 10% neutral-buffered formalin, samples must be rinsed under running water to remove formalin.

The work sequence is alcohol 70% for 30 min, alcohol 95% I° for 2–3 h, alcohol 95% II° overnight, alcohol 100% I° 2–4 h, alcohol 100% II° 2–4 h, xylene I° 2–4 h, xylene II° overnight, paraffin I° 2–4 h, paraffin II° 2–4 h, in the oven at 56–58°C.

As the specimens of the brain stem are fairly large, it is advisable to use metal molds as for the conduction system.

**Figure 13:**
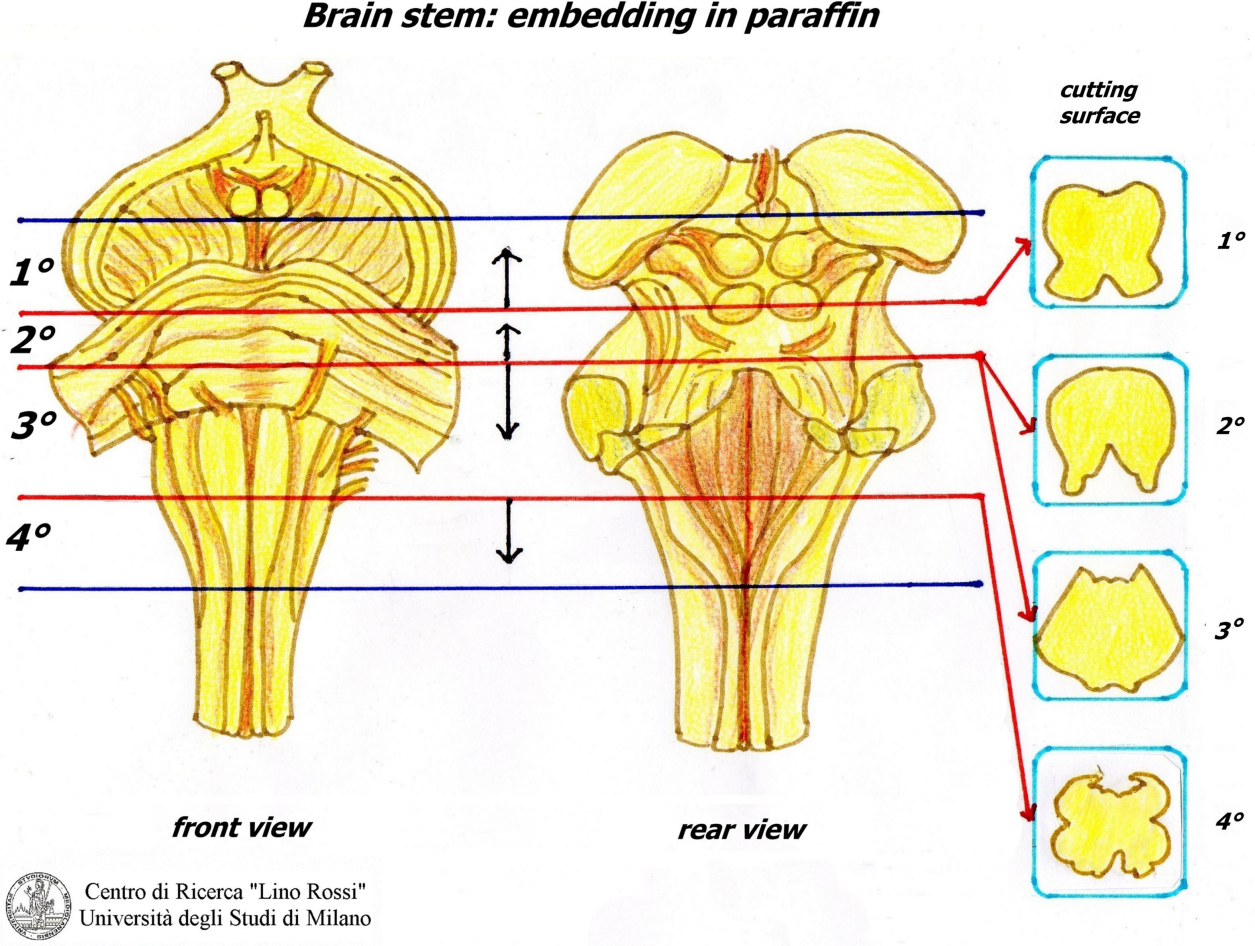
**Orientation of brainstem samples**.

The first block is embedded from the pons; the second block is embedded from the caudal surface; however, it is not cut serially if not required; the third block is embedded from the pons; the last block is embedded from the cranial surface (Figure 13).

In this way, all the samples are embedded, as it is essential to include all of the anatomical structures.

The samples are embedded with the aim of achieving the control of vital functions before the main structures.

The thoracic spinal cord is excised from the tract between T1 and T5.

It is cut in slices, which are processed together with the brainstem, but with shorter processing times.

The slices of the thoracic spinal cord are put between two sponge pads for biopsy that are then placed in mega-cassettes, in order to avoid movement.

**Figure 14:**
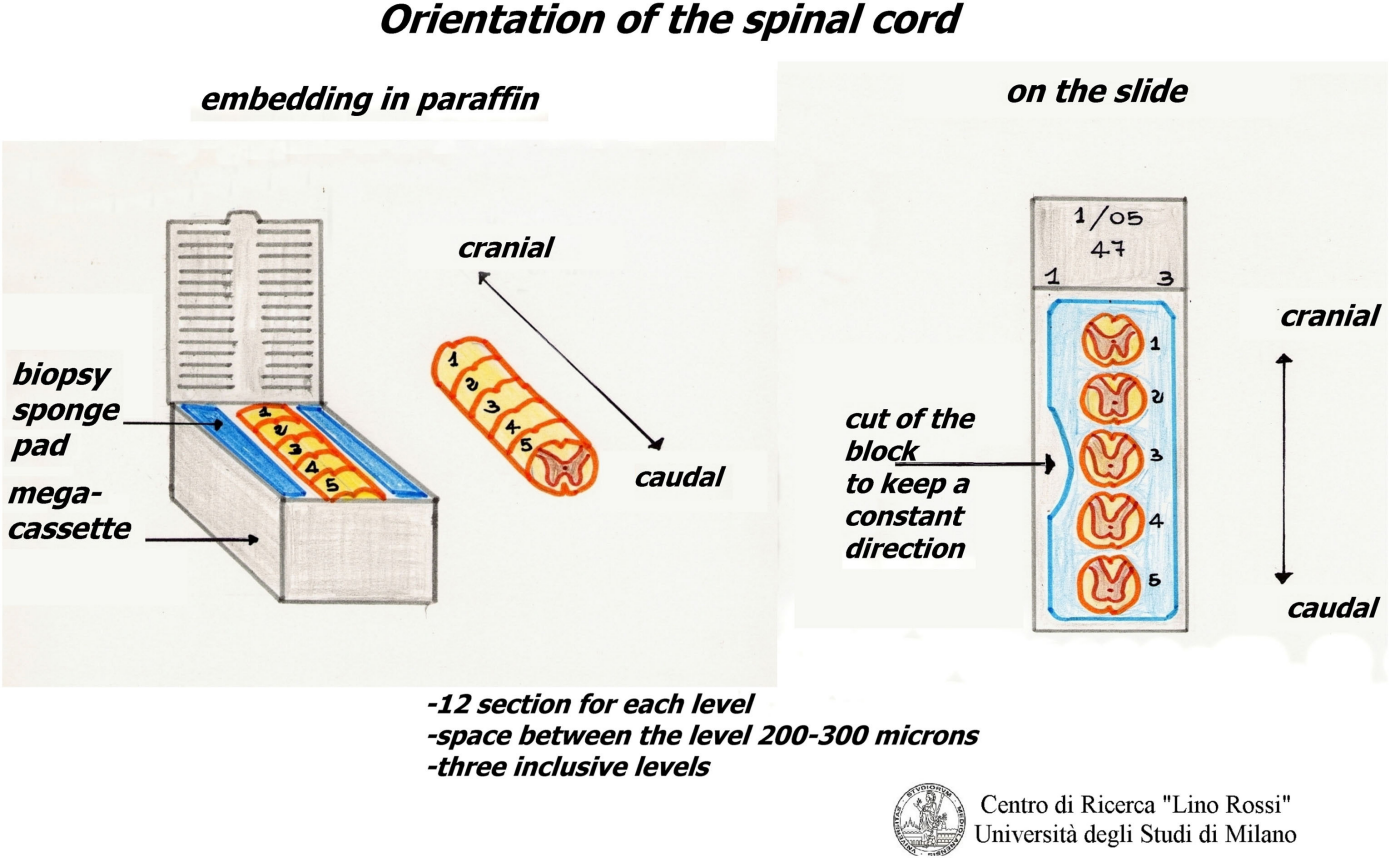
**Embedding of thoracic spinal cord**.

The slices are embedded in the same way, in the correct orientation, for an accurate reconstruction (Figure 14).

This procedure helps to reduce the number of slides.

The cutting protocol for the brainstem is as follows: first, the block is cut for DNA extraction; the sections are prepared with a blade cleaned with xylene in order to avoid contamination, while the technician, wearing gloves, cuts six 5 μm thick sections and places them in well-sealed sterile test tubes.

The brainstem serial section is carried out without intermediate intervals, apart from those required for adjusting the surface after necessary interruption.

First, all the blocks are cut for 15 levels each. Following a preliminary examination, it is possible to cut them again, until all the nervous centers have been sliced.

Each level requires 12 6 μm thick sections (4 normal blank and 8 on silanized slides). All of them must be carefully marked and placed in an oven heated to 37°C overnight.

The following day the H.E. sections are screened, one for each level, subsequently further methods will be decided.

This protocol can also be used for the thoracic spinal cord, but only on three levels, and material ranging between 200 and 300 µm in size is discarded from one level and the next.

Klüver-Barrera (KB) (1953) is the most suitable staining for the brainstem. It is carry out with Luxol Fast Blue MBS (Color Index Solvent Blue 38, Sigma-Aldrich, code S3382) which is generally used for staining myelin fibers; the dye is classed as a solvent (oil-soluble) dye, which links components such as lecytin and sphingo-myelin, i.e., phospholipidic myelin substances.

The Luxol counterstain is provided by the Cresyl Violet (Sigma-Aldrich, code C5042), which enables us to obtain a clear image of the nuclear chromatin.

Klüver-Barrera was used for taking morphometric measurements at each suitable level (27).

For highlighting tigroid substance and ribonucleic acid, the best results are obtained with the Cresyl Violet method.

Another popular staining technique is the silver impregnation, Bielschowsky’s method, which is used for demonstrating neurons, axons and dendrites by staining them black. First, the sections are impregnated with silver nitrate salts and the deposit is intensified by adding a solution containing silver nitrate and formalin as a reducing substance. Tone with gold chloride and fix with sodium thiosulphate, as the final step.

**Figure 15:**
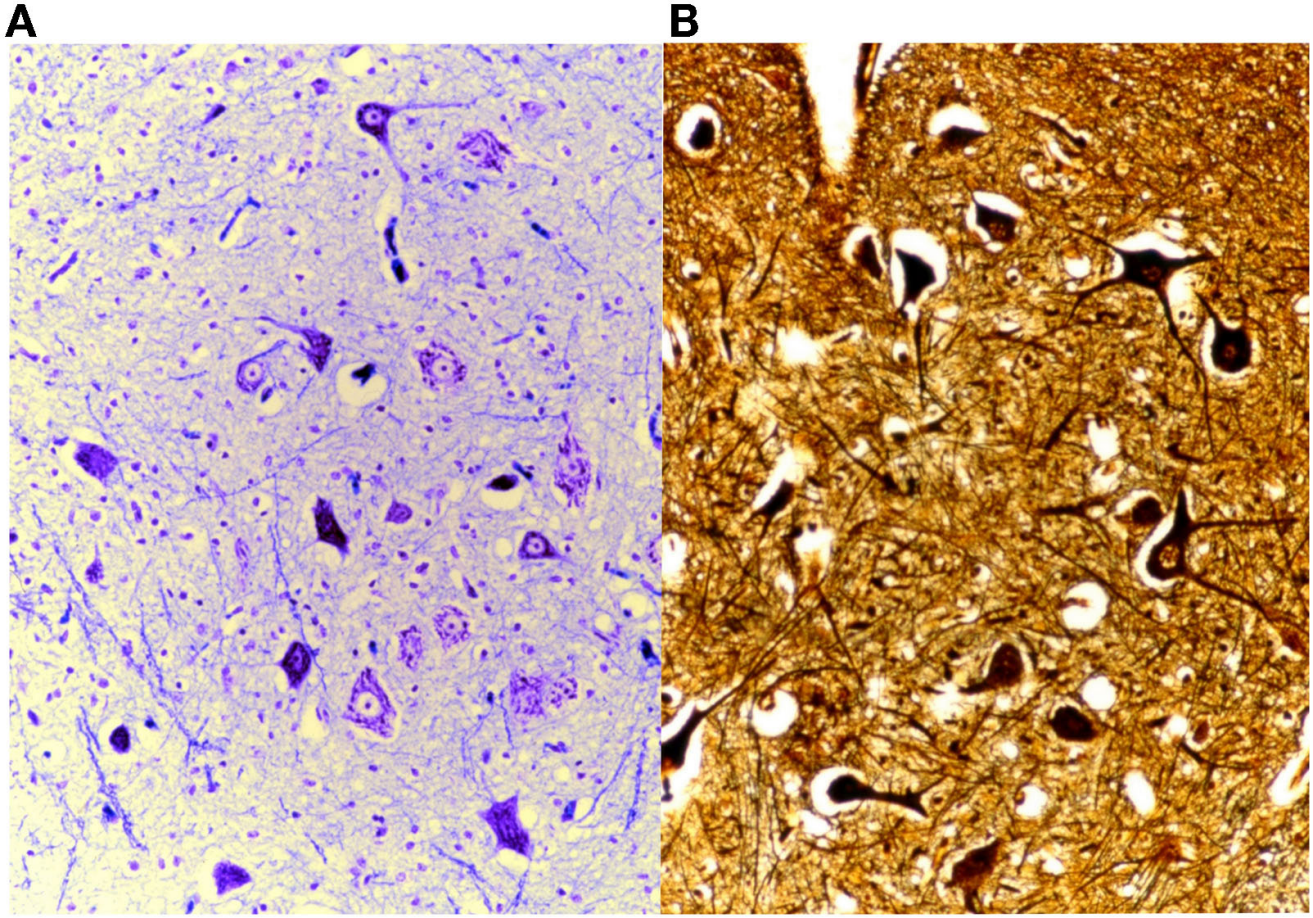
**On the left (A), brain stained with Klüver-Barrera, 25× and, on the right (B), Gless-Marsland, 25×**.

Another of our methods is the silver impregnation by Gless-Marsland, which is particularly suitable for the brain stem, which stains the neuronal bodies and the neurofibres black or brown, on a lighter bronze-brown background (Figure 15).

As already mentioned, Mallory’s P.T.A.H. (see above) can be used to stain the glia satisfactorily.

Other staining materials are Lillie for the neuromelanin pigment (28) and Perl’s Prussian blue for iron in oxygen-transporting heme mechanism (29).

While for apoptosis, the blank slides must be kept in an oven heated to 60°C for 4 h prior to performing the reaction.

Substances, such as somatostatin (Abcam, ab108456), substance P antibody [DBA, Segrate (MI), Italy, SP, C 191], and tyrosine-hydroxylase (Abcam, ab75875), PHOX2B (H-20) gene (Santa Cruz Biotechnology, SC 13224), and NeuN antibodies (Millipore, S 7100), are used for some of the immune-histochemical reactions (30–32).

Most of the staining solutions are made from basic commercial substances within the laboratory, and the blank slides proposed, correctly labeled and archived, are available upon request for carrying out further staining using appropriate histo- and/or immunochemical techniques.

## Expected Results

The regulations of Mortuary Police; paragraph of statute 8, 9, 10 (Italian Law 2 December 1975, no. 644) foresees that “no dead body … may be subjected to autopsy … earlier than 24 hours from the time of death” (33).

This means that the body degenerative events occur as autolysis, self-digestion, and putrefaction by microorganisms.

However, the autopsy is carried out in time, and the chemical fixation of the samples is performed immediately, only slight damage is caused by degenerative phenomena.

Following the technical protocols described in this paper, it should ensure an excellent outcome of the histological slides.

The best way of preserving anatomical structures is through timely and prolonged fixation, manual processing with variable times according to the size of the samples, thickness of sections, and hand-made stainings.

Unfortunately, the damage caused by lack of fixation is irreparable, as in the case of those fetuses who die in the womb.

Inside the heart, large blood clots develop within the cardiac chambers. As soon as possible, before sampling, it would be advisable to open the heart by cutting the tip and cleaning it out.

Lysis phenomena, partial or total degeneration, and bacterial infiltration, up to complete loss of morphological details can occur very severely in the brain, as well as other organs.

These histological slides show the complete loss of morphology and affinities of the dyes, the immune-histochemical reactions are negative and/or diffuse.

Brain liquefaction may occur leading to incomplete diagnosis.

## Conclusion

In the event of SIDS or SIUDS, a large number of slides are required in order to carry out an accurate pathological investigation.

A complete case requires approximately 2100 slides of which 488 stained and 1612 blanks, while 1272 slides are require for an incomplete case, including 274 stained.

Approximately 3–4 weeks are generally required to prepare each case.

A large number of slides are required for diagnosis and research: this means that these protocols enable us to carry out in-depth studies on anatomical structures.

The slides are performed before a case is found to be of interest.

The technique is simple but lengthy in the preliminary phase.

For this purpose, a laboratory and specialized technicians are required for manual processing, which differs according to the type of sample. Considerable knowledge is required for this task as well as automated routine techniques.

Using stains made in the laboratory helps to lower costs but it foresees collective and individual protection.

The technician has to seek and love the “perfect slide.”

## Author Contributions

GA wrote the text of the article and MC contributed to the graphic, drawing the figures. All authors listed, have made substantial, direct and intellectual contribution to the work, and approved it for publication.

## Conflict of Interest Statement

The authors declare that the research was conducted in the absence of any commercial or financial relationships that could be construed as a potential conflict of interest.
